# Supplementary material for: Venous Thromboembolism and Its Risk Factors in Children with Acute Lymphoblastic Leukemia in Israel: A Population-Based Study
Source: Cancers (Basel). 2020 Sep 25;12(10):2759. doi: 10.3390/cancers12102759 (PMC7600511; doi:10.3390/cancers12102759)
Supplement: Supplementary file 1 [file cancers-12-02759-s001.pdf]

Venous Thromboembolism and Its Risk Factors in Children with Acute Lymphoblastic Leukemia in Israel: A Population-Based Study

Shlomit Barzilai-Birenboim, Ronit Nirel, Nira Arad-Cohen, Galia Avrahami, Miri Ben Harush, Assaf Arie Barg, Bella Bielorai, Ronit Elhasid, Gil Gilad, Amos Toren, Sigal Weinreb, Shai Izraeli and Sarah Elitzur

Table S1. Characteristics of patients with ALL by the hospital (*n* = 1191).

Characteristic	Total *n* = 1191	Bnai Zion *n* = 24	Dana *n* = 67	Soroka *n* = 100	Hadassah *n* = 177	RR *n* = 196	SCMCI *n* = 388	Sheba *n* = 239	*p*-Value *
Gender									
Female	501	9	37	37	82	81	151	104	0.13
Male	690	15	30	63	95	115	237	135	
Age group									
<10 Years	840	18	49	69	131	133	272	168	0.88
>10 Years	351	6	18	31	46	63	116	71	
Risk group									
HR	262	4	13	18	37	48	86	56	0.84
nHR	929	20	54	82	140	148	302	183	
Lineage									
T	190	4	12	13	30	36	70	25	0.17
B	1001	20	55	87	147	160	318	214	

* Fisher exact test for the hospital; Abbreviations: HR, high risk; nHR, non-high-risk; SCMCI, Schneider Children's Medical Center; RR, The Ruth Rappaport Children's Hospital.

Table S2. Consensus definition of thromboembolism, Ponte Di Legno Working Group (PTWG)25.

Grade.	Definition
1	Superficial thrombophlebitis or central venous line-associated deep vein thrombosis without symptoms (e.g. swelling, discoloration, or collaterals), or causing only central venous line dysfunction (systemic anticoagulants not given)
2A	Asymptomatic thromboembolism including asymptomatic cerebral thrombosis. Systemic anticoagulation is usually given (not evidence-based)
2B	Symptomatic deep vein thrombosis, systemic anticoagulation indicated
3	Symptomatic pulmonary embolism, cardiac mural thrombus without cardiovascular compromise, symptomatic cerebral sinovenous thrombosis, or arterial ischemic stroke; all require systemic anticoagulation/antiaggregating
4	Life-threatening thromboembolism, including arterial insufficiency, hemodynamic or neurological instability; urgent intervention needed
5	Death due to thromboembolism

Confirmation of venous and/or arterial thromboembolism: by imaging (ultrasonography, computed tomography, magnetic resonance imaging) or by autopsy is required for grade 2 and higher.

Table S3. Characteristics of children with cerebral sinus vein thrombosis.

Children Characteristics	Children with CSVT *n* = 21 (1.8%)	Children without VTE *n* = 1102	*p*-Value
Median age years (range)	10.3 (1.7–18.5)	7.3 (1–19.3)	<0.001 *
CNS status at diagnosis			
CNS 1	19 (90.4%)	1035 (93.9%)	0.21+
CNS 2	1 (4.8%)	38 (3.5%)	
CNS 3	1 (4.8%)	29 (2.6%)	
Mortality	1 (4.8%)	87 (7.9%)	0.77+
Relapse	2 (9.5%)	71 (6.4%)	
Therapy phase of CSVT *n* (%)			
Induction	18 (85.7%)		
Consolidation	2 (9.5%)		

Delayed intensification	(4.8%)
Symptoms of CSVT	
	Severe symptoms (*n* = 18)<br>Median age of 11.3 years (range: 1.7–18.5)<br>Seizures *n* = 8 (38.9%)<br>Hemiparesis +/− facialis *n* = 5 (23.8%)<br>Severe headaches *n* = 3 (14.3%)<br>Coma *n* = 2 (9.5%)<br>Moderate symptoms (*n* = 3)<br>Median age of 6.2 years (range 4.1-9.0)<br>Moderate headaches +/− irritability *n*= 2 (9.9%)<br>Earaches *n* = 1 (4.8%)

Abbreviations: CSVT, cerebral sinus vein thrombosis; CNS, central nerve system; * Wilcoxon-Mann-Whitney; + Fisher's exact test; Children without VTE, *n* = 89; Children with mild VTE, *n* = 65; with CSVT, *n* = 21; with PE, *n* = 3.

Table S4. Common Terminology Criteria for Adverse Events (CTACE), version 4.03-Hypertriglyceridemia.

Hypertriglyceridemia *				
**Grade 1**	**Grade 2**	**Grade 3**	**Grade 4**	**Grade 5**
150–300 mg/dL; 1.71–3.42 mmol/L	>300–500 mg/dL; >3.42–5.7 mmol/L	>500–1000 mg/dL; >5.7–11.4 mmol/L	>1000 mg/dL; >1.4 mmol/L; life-threatening consequences	Death

* Definition: A disorder characterized by laboratory test results that indicate an elevation in concentration of triglyceride concentration in the blood.

Table S5. Triglyceride levels and VTE events during two BFM protocols.

**Triglycerides**	**All** *n* **= 584**	**BFM 2002** *n***-274**	**BFM 2009** *n***-310**	**No VTE** *n* **= 535**	**Any VTE** *n* **= 49**	**Severe VTE** *n* **= 18**	**Mild VTE** *n* **= 31**
TG < 500 mg/dL	394 (67.5%)	199 (72.6%)	195 (62.9%)	373 (69.7%)	21 (42.8%)	7 (38.9%)	14 (45.2%)
TG 1000-500 mg/dL CTCAE grade 3	123 (21.1%)	51 (18.6%)	72 (23.2%)	106 (19.8%)	17 (34/7%)	4 (22.2%)	13 (41.9%)
TG ≥ 1000 mg/dL CTCAE grade 4	67 (11.5%)	24 (8.8%)	43 (13.9 %)	56 (10.5%)	11 (22.4%)	7 (38.9%)	4 (12.9%)
Severe TG ≥ 500 mg/dL CTCATE grade 3 + 4	190 (32.5%)	75 (27.4%)	115 (37.1%)	162 (30.3%)	28 (57.1%)	11 (61.1%)	17 (54.8%)

Measured in SCMCI (Schneider Children's Medical Center of Israel) and RR (The Ruth Rappaport Children's Hospital); Abbreviations: TG, triglycerides; VTE, venous thrombosis; *p* values for children with/without severe hypertriglyceridemia (TG ≥500 mg/dL CTCAE grade 3 + 4): Any VTE vs no VTE; *p* = 0.001 (Fisher exact test.);Mild vs no VTE; *p* = 0.010 (Pairwise analysis); Severe vs no VTE; *p* = 0.006 (Pairwise analysis); TG levels between protocols; *p* = 0.013 (Fisher exact test.).

Table S6. Relapse rate and mortality in patients with/without VTE events *p* = N.S.

**VTE**	**CR** *n* **(%)**	**Death** *n* **(%)**	**Relapse** *n* **(%)**	**LFU** *n* **(%)**	**Total**
No	933 (85%)	88 (8%)	72 (7%)	9 (1%)	1102
Yes	72 (81%)	5 (6%)	8 (9%)	4 (4%)	89
Total	1005 (84%)	93 (8%)	80 (7%)	13 (1%)	1191

p-value of pairwise by Fisher exact tests: CR-Death: *p* = 0.67; CR-Relapse: *p* = 0.37; Death- Relapse: *p* = 0.25; Abbreviations: CR, complete remission; LFU, lost to follow-up; VTE, venous thrombosis.

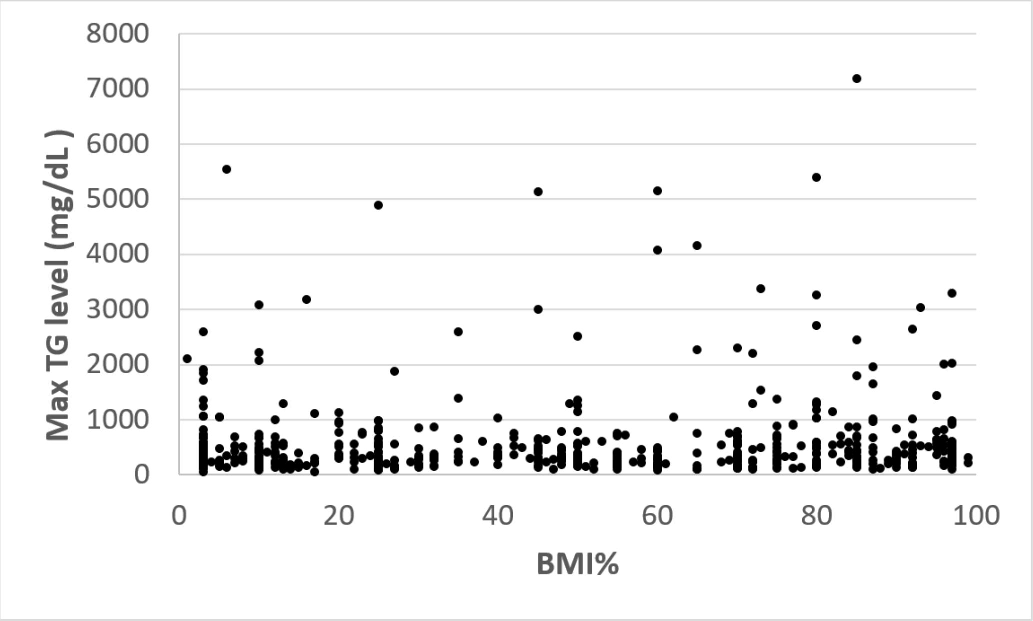

Figure S1. Lack of association between BMI% and maximal TG level (*n* = 548 *). Pearson correlation = 0.08; * Evaluated for all children from two tertiary medical centers (SCMCI; Schneider Children's Medical Center of Israel and RR; The Ruth Rappaport Children's Hospital); Abbreviations: TG, triglycerides.

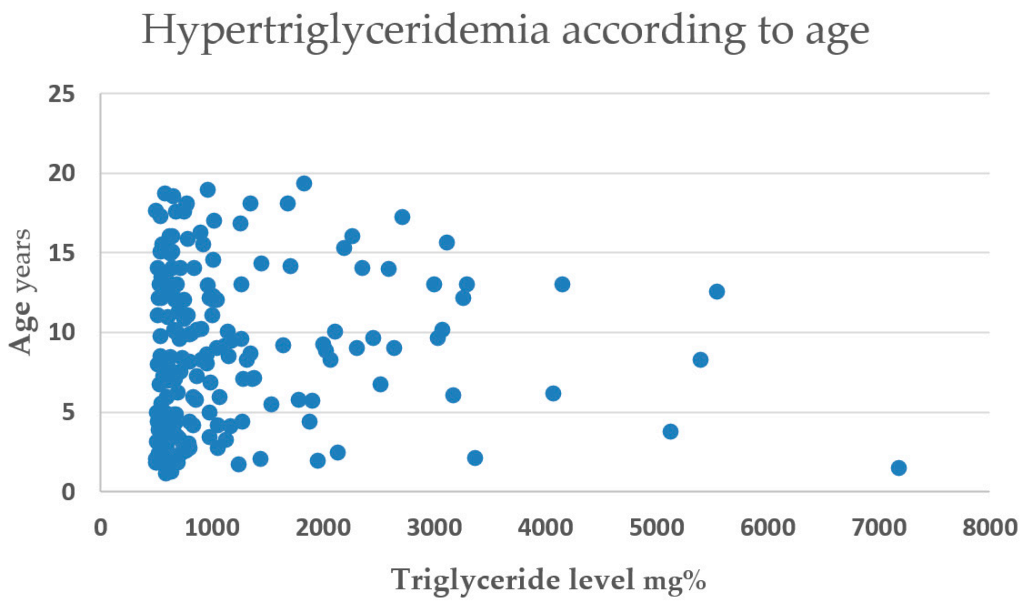

Figure S2. Lack of association between hypertriglyceridemia and age (*n* = 548 *). Pearson correlation = 0.05, * Evaluated for all children from two tertiary medical centers (SCMCI; Schneider Children's Medical Center of Israel and RR; The Ruth Rappaport Children's Hospital), Abbreviations: TG, triglycerides.

© 2020 by the authors. Licensee MDPI, Basel, Switzerland. This article is an open access article distributed under the terms and conditions of the Creative Commons Attribution (CC BY) license (http://creativecommons.org/licenses/by/4.0/).
